# Are only-children different? Evidence from a lab-in-the-field experiment of the Chinese one-child policy

**DOI:** 10.1371/journal.pone.0277210

**Published:** 2022-11-08

**Authors:** Fredrik Carlsson, Elina Lampi, Peter Martinsson, Qin Tu, Xiaojun Yang

**Affiliations:** 1 Department of Economics, University of Gothenburg, Gothenburg, Sweden; 2 Department of Technology, Management and Economics, Technical University of Denmark, Copenhagen, Denmark; 3 Center for Innovation and Development Studies, Beijing Normal University at Zhuhai, Zhuhai, China; 4 School of Public Policy and Administration, Xi’an Jiaotong University, Xi’an, China; Universität Heidelberg, GERMANY

## Abstract

In this paper, we present evidence from a lab-in-the-field experiment of the effects of the Chinese one-child policy on adults in China who were born just before and after the introduction of the policy. We measure risk, uncertainty, and time preferences, as well as subjects’ preferences in the social domain, i.e., concerning competitiveness, cooperation, and bargaining. We sampled people from three Chinese provinces born both before and after the introduction of the policy in 1979. We utilize the fact that the one-child policy was introduced at different times and with different degrees of strictness in different provinces. Overall, we find a statistically significant effect only on risk and uncertainty aversion and not on any other preferences in the experiments: Those born after the introduction of the one-child policy are less risk and uncertainty averse. These results hold for various robustness checks and heterogeneity tests. Hence, our results do not confirm the general wisdom and stereotype of only-children in China being “little emperors.”

## 1. Introduction

It is important to understand what shapes people’s preferences since preferences affect behavior and hence success in life. It is widely believed that being an only-child has long-term effects on behavior, especially in terms of being more selfish and less cooperative, but the empirical evidence is mixed on this topic [see, e.g., 1, 2]. For obvious reasons, it is almost impossible to test any family policy related to number of children since the number of children is not random, although one can use twin births or other random events as instruments [3–5]. One way to remedy this problem is to use family planning campaigns and policies as exogenous variables to investigate the effect of the number of children in a family on the future behavior of these children. China’s one-child policy (OCP), introduced in 1979, is one of the most well-known family policies of all time and offers an opportunity to investigate the effect of being an only-child.

The objective of this paper is to explore whether being an only-child affects preferences, and we focus our research on risk and time preferences and preferences in the social domain (competitiveness, cooperation, and bargaining). We exploit the OCP as our identification strategy. We conduct lab-in-the-field experiments among adults in China who were born just before or after the introduction of the OCP in 1979, and we utilize the fact that the OCP was introduced at different times in different provinces in order to identify the effect.

According to Heckman and Kautz soft skills such as personality traits and preferences are important for success in life in general, including in the labor market [6]. There is a vast literature, mostly in sociology and psychology, on differences in pro-social behavior and in cognitive and personality traits between children brought up with and without siblings [7]. The general belief and stereotype are that only-children are “little emperors,” since they are believed to receive undivided attention from their parents and grandparents, while at the same time facing strong expectations to excel in life. However, the empirical findings on the behavioral effects of being an only-child are mixed. In a meta-analysis summarizing 115 studies on only-children in China and the United States, Falbo reported no support for the negative stereotypes commonly attached to being an only-child [2]. Instead, the opposite was found for many outcomes and characteristics. Finally, Chen and Goldsmith reviewed a large number of studies on only-children and their behavior and concluded that the findings are inconsistent and inconclusive [1].

The impacts of being an only-child are especially interesting to study in China, where a large share of the population have been only-children with peers who have also been only-children since the introduction of the OCP in 1979. Today, 25% of all families in China have only one child, meaning that there are more than 100 million only-children [8]. Moreover, the OCP has resulted in the largest sex-ratio imbalance in the world, with around 1.2 males per female [9–11]. Moreover, the regions with a stricter OCP and higher fines for unauthorized births have even higher male-to-female ratios [12]. The empirical findings on being an only-child have been inconclusive in the context of China: Peng found that only-children are more self-centered [8], Shen and Yuan found no evidence that only-children in China are more “spoiled” [13], and Falbo and Poston concluded that the OCP has not created a generation of “little emperors” [14]. Moreover, Wang et al. did not find that only-children more often have undesirable personality traits than children with siblings [15]. There is also a literature concerning the effect of being an only-child on mental wellbeing. Tseng et al. found that female only-children are more likely than male only-children to experience depression [16]. On the other hand, Yang et al. found that only-children in China are actually better off as adolescents, having lower levels of fear, anxiety, and depression [17]. Zhang et al. asked parents to freely describe their children and found that although the little emperor syndrome might exist in a preschool-aged only-child, it often gradually disappears as the child becomes older [18].

Cameron et al. investigated the impacts of the OCP on the behavior of adults [19]. They conducted four economic experiments together with a survey to elicit personality traits among individuals born before (1975–1978) and after (1980–1983) the introduction of the OCP in 1979, and found that people born after the introduction of the policy have lower levels of trust and are less trustworthy, more risk-averse, less competitive, and more pessimistic, though not less altruistic. Thus, their results confirm the common view about the negative impacts of the OCP on preferences and behavior. Moreover, they found that these impacts are long-lasting since the subjects were in their 30s at the time of the experiment. Other studies in economics have found that only-children in Sweden are more competitive and status-driven than others, but this is in a context without restrictions on family planning [20].

When analyzing the effect of the OCP, it is important to recognize that the policy was less stringent when first introduced in 1979 (henceforth referred to as the first stage of the OCP), but then became stricter in terms of enforcement and punishment over time (referred to as the second stage of OCP). Moreover, the OCP was implemented at different times in different provinces, and the intensity in enforcement and punishment varied as well, especially during the second stage [21]. Previous literature has used this variation in timing across provinces to estimate the impact of the OCP on various family outcomes [22] and education [23, 24]. In line with the literature, the present study also utilizes the fact that the OCP was introduced at different times and with varying intensity across provinces. We therefore conducted our study in three cities–Guilin, Wuxi, and Lanzhou–all of which are located in different provinces and differ in OCP timing. We selected these cities with great care in order to reduce the risk of confounding effects from provincial differences in OCP. To this end, we defined three important criteria for our sampling frame: (i) being Han Chinese since the policy differed for other ethnic groups, (ii) being born 1976–1986 to allow for pre- and post-analyses of OCP implementation, and (iii) being born and raised in the same province. We believe that these three criteria allow for a clean test of the effects of the OCP and being an only- child on preferences. In addition to risk and competitiveness experiments, we also investigate three types of preferences (time, bargaining, and cooperation) that Cameron et al. did not address [19]. Thus, we believe that we also cover previously not studied behavioral aspects considering the possible “myth” of self-centered only-children.

Our paper contributes to the literature on understanding the effect of being an only-child and the effect of the OCP in China. The study by Cameron et al. was conducted in Beijing only [19]. Beijing differs from most other parts of China in terms of the OCP, as the strict implementation of the family planning policy in fact began before 1979, resulting in low fertility rates already before 1979 [25]. Whether the effects of being an only-child (due to the OCP) on preferences is larger in Beijing than in other cities depends, among other things, on the difference in OCP strictness between Beijing and other cities.

The rest of the paper is organized as follows: Section 2 presents the family planning policies in China. A description of our experimental design is provided in Section 3. Section 4 presents the descriptive statistics, and the results are given in Section 5. Finally, Section 6 discusses our results and concludes the paper.

## 2. Family planning policies in China

### 2.1. The national policies

Already in the mid-1950s, Chinese authorities had initiated a first family planning campaign with the goal of reducing the country’s population. A second campaign started in the 1960s, but it was suspended in connection with the Cultural Revolution. A third was launched in the early 1970s, known as the “later, longer, and fewer” campaign, which encouraged people to get married later in life, have fewer children, and have larger age gaps between the children [8]. The third campaign resulted in a sharp reduction in fertility rates, from 5.9 children per women in 1970 to 2.7 in 1979 [26].

In October 1978, the central government explicitly advocated the policy “one is best, two at most” and that siblings should be born at least three years apart. In June 1979, the compulsory family planning campaign was launched at the second meeting of the fifth People’s Congress. We define this as the first stage of the OCP. The OCP included several types of penalties for having more than one child, but the penalties and when they were implemented varied among provinces. Examples of penalties include various health care and schooling restrictions for the second child and monetary punishments for parents in the form of withdrawn bonuses at work or no wage increases. For government employees and Communist Party members, additional political and disciplinary punishments were implemented, such as not being able to be promoted politically, and government employees could lose their jobs. Conversely, families with only one child received economic support.

Although the OCP was specified as a national policy for the whole country, its enforcement varied in response to local sociodemographic and economic conditions [11, 22]. In rural areas, people were allowed to have a second child if the first child was a girl and the age gap between the two children was at least four years. Furthermore, there were even fewer or no restrictions on families from ethnic minorities. The “family planning” was stipulated as the basic national policy at the twelfth meeting of the Chinese Communist Party in 1982.

### 2.2. Implementation of the OCP in the three sampled cities

We conducted our study in three cities: Guilin in the province of Guangxi (southern China near Guangzhou), Wuxi in the province of Jiangsu (eastern China near Shanghai), and Lanzhou in the province of Gansu (western China near Xi’an). Table 1 summarizes the basic characteristics of the three cities. All three cities are large, but Wuxi is more developed than the other two, which explains the higher gross domestic product (GDP) per capita. The differences in mean urban disposable annual income per capita and average annual salary are smaller.

**Table 1:** Characteristics of the three sampled cities.

Variable	Guilin	Wuxi	Lanzhou
Metropolitan area (km^2^)	565	1643	1574
Resident population in metropolitan area	1,507,200	3,613,800	2,659,700
GDP per capita (yuan)	34,844	126,389	54,771
Urban disposable annual income per capita (yuan)	26,811	41,731	23,030
Urban average annual salary (yuan)	45,194	67,048	51,928

*Source*: The data come from Guilin [27], Wuxi [28] and Lanzhou [29] (statistical yearbooks in 2015).

As discussed above, before the introduction of the OCP in 1979, several family planning campaigns had been launched in the mid-1950s. According to Zhang [22], the decrease in fertility was significantly smaller after the introduction of the OCP than during the early 1970s: the country’s total fertility rate declined from 5.8 in 1970 to 2.7 in 1978, and the corresponding decline from 1978 to 1995 was from 2.8 to 1.8 children per woman. At the time the policy was introduced, fertility rates were decreasing in urban areas in all three studied provinces, and the drop in fertility rates was even larger in our three studied provinces than in Beijing (per our own calculations based on Coale and Li [25]). Although the three sampled cities implemented the national family planning policy around the same time in 1979, the timing of the implementation of the stricter OCP varied because of the different local administrative processes, this is the basis of our identification strategy. Based on information from city family planning policy archives and chorography, we next summarize the implementation of the OCP in each of the three studied cities.

#### 2.2.1. Guilin

On September 20, 1979, the Guilin municipal government issued a document, titled The Provisional Regulations on Family Planning. This document stipulated the basic requirement of “one is best, two at most,” with at least three years between the first and second child. The OCP became stricter with a document, issued on May 18, 1981, titled Supplementary Regulations on Family Planning and Control Population Increase. It clearly specified that each couple could have only one child. For families with more than one child, the parents’ salary would be reduced by 10% until the second child was 7 years old, and the reduction would increase for each additional child. In contrast, couples who had only one child would be rewarded with childcare, medical services, parental leave, and pensions.

#### 2.2.2. Wuxi

On July 31, 1979, the Wuxi government introduced the “one is best, two at most” policy. From May 1, 1980, couples who had three or more children (not including twins in the second birth) had to pay fines. From June 1982, Wuxi followed the provincial policy that government officials, employees, and citizens could have only one child. For families with more than one child, the parents’ salary would be reduced by 10% for 7 years for the second child and 20% for 10 years for the third child. In addition, other welfare benefits would be suspended for couples with more than one child, including medical services, salary during parental leave, and opportunities to be promoted.

#### 2.2.3. Lanzhou

On July 14, 1979, Lanzhou adopted the provincial family planning policy, “one is best, two at most.” Couples who had three or more children had to pay extra child fees. On 20 April 1982, the Lanzhou government issued supplementary announcements about implementing a provincial document titled The Regulations on the Specific Policies of Family Planning. Urban citizens could have only one child. If a family had a second child without being exempt from the rule, the mother’s salary would be temporarily suspended, and both the father’s and the mother’s salaries would be reduced by 10% until the child reached the age of 10. The fines would increase with the number of additional children a family had.

## 3. Experimental design and procedure

### 3.1. Experimental design

We investigate risk preferences, time preferences, and preferences in the social domain. More precisely, the first two experiments concerned risk and time preferences, and the others concerned social behavior in a setting with strategic interaction: competitiveness in a tournament experiment, cooperation in a public good experiment, and bargaining in an ultimatum bargaining experiment. All experiments followed standard designs, but since they were implemented in the field, some modifications were made, especially considering logistics issues. The five experiments are described in detail in the Supporting Information (S1 Text). We briefly present them below.

In the risk experiment we elicited preferences for lotteries with known probabilities of winning of 10%, 50%, and 90%. We used a choice list where subjects chose between a safe amount and a lottery with a possibility of winning a fixed amount (80 yuan) [30]. Subjects made multiple choices and for each new row of the choice list, the safe amount was gradually increased from 1 yuan to 80 yuan. We measure risk preferences based on the point in the list where a subject switched from choosing the lottery to choosing the safe amount. Thus, the earlier a subject made this switch, the more risk averse (or less risk loving) they were considered to be. Subjects were told that once they switched to the safe amount, they were not allowed to switch back to the lottery option later in the list of decisions. To make it a bit easier for the subjects, they were instructed to draw a line between the rows where they started to prefer the safe amount. The risk experiment was implemented by using a bag, and inside the bag there are 10 balls numbered 1 to 10. In the case of 10% winning probability a subject bet on a number between 1 and 10 and then picked a ball. If the guess and the number of the ball matched, then the subject won. In addition to the risk preference experiment, which is characterized by known probabilities, we also elicited preferences for the case of unknown probabilities (uncertainty). The experiment with uncertainty was similarly designed, but the distribution of numbers on the balls was unknown. With a 10% winning probability, the subjects were told that there were many balls in a bag numbered from 1 to 10 but that the distribution of the numbered balls was unknown. They were then asked to bet on one number.

In the time preference experiment, subjects were asked to make repeated choices between a sooner, fixed payment (40 yuan) and a later payment that would increase from 41 yuan to 60 yuan. We were interested in the switching point from a sooner to a later payment in four different tasks where the subjects had to make decisions between (i) today and in one week, (ii) in one week and in two weeks, (iii) today and in two weeks, and (iv) in two weeks and in four weeks. As in the risk and uncertainty experiments, the subjects were told that once they chose a later payment option, they had to continue choosing that option throughout the rest of the list. To make it a bit easier, subjects were instructed to draw a line between the rows where they started to prefer a later payment.

The experiment on competitiveness followed the design of Niederle and Vesterlund [31]. Subjects completed three tasks, but only one would be randomly selected as payoff relevant. Each subject was randomly matched with three other participants to form a group, but they did not know who the other group members were. The group composition remained the same throughout the competitiveness experiment and, in each group, there were two men and two women. In the experiment, subjects were to calculate the sum of five randomly chosen two-digit numbers. There were three tasks. Task 1 was paid piece rate, i.e., subjects were paid 3 yuan per problem solved if the task was randomly selected for payment. Task 2 was a tournament in which subjects had three minutes to solve the same type of math problems. The group member who solved the largest number of problems received 12 yuan per correct solution, while the other participants received no payment. For Task 3, subjects first had to choose a payment schedule, piece rate or tournament, and then solved the same type of math problems. If a subject chose tournament, their performance was evaluated relative to the performance of the other three group members in Task 2.

The one-shot public good experiment used a strategy design similar to that of Fischbacher et al. [32]. Each subject was endowed with 20 tokens, each token being equivalent to 2 yuan. Each unit invested in the public good generated an income of 0.4 for each of the four group members, creating a conflict between the private and social optimum. Subjects made two contribution decisions in a fixed order: first unconditional and second conditional. In the unconditional decision, subjects decided how many tokens to invest in a public good. In the conditional decision, subjects decided how much to contribute to a public account conditional on a specific average contribution of the other group members.

In the ultimatum bargaining experiment, subjects were randomly matched in pairs as Player 1 and Player 2. Subjects did not know their roles beforehand, so they had to make their decisions as both Player 1 and Player 2. The experiment worked as follows: Player 1 decided how to allocate an endowment of 40 yuan between the two subjects, and Player 2 decided whether to accept or refuse the allocation. If Player 2 accepted Player 1’s allocation, then Player 1 and Player 2 split the money according to Player 1’s allocation. If Player 2 refused Player 1’s allocation, then neither player received anything. The order of the roles was not randomized, instead, all subjects first answered as Player 1 and then as Player 2.

### 3.2. Experimental procedure

We conducted the experiments in Guilin in June 2014, in Wuxi in November 2014, and in Lanzhou in December 2014. Our study was conducted with the verbal permissions by 1) Guilin Municipal Health and Family Planning Commission; The local government of sample districts including Diecai, Qixing, Xiufeng and Xiangshan.2) Wuxi Municipal Health and Family Planning Commission; The local government of sample districts including Beitang, Chongan and Nanchang. 3) Lanzhou Municipal Health and Family Planning Commission; The local government of sample districts including Chengguan, Anning and Qilihe. To identify and analyze the direct effects of the OCP, we aimed at a sampling frame that included only people who were born in 1976–86 in the sampled city, whose parents had an urban hukou (residence) at the time of their birth, and who belonged to the Han majority. Hukou refers to the Chinese household registration system. Since the policy was only strictly applied to people with a city hukou, we had to make sure that subjects’ parents had a city hukou. When defining the frame, we needed to consider available register data as well as what was practically and logistically feasible. Thus, the selection of subjects was done in several steps. First, we used the community registration system, which contains lists of all households in the city. These lists also include people not eligible for our study, and hence we needed to remove them before making a random selection of participants. Since the cities are large (1.5–3.6 million inhabitants), it was not practical to go through all households in the register to create a sampling frame from which we would randomly select subjects. Instead, we drew a random sample from the register and then asked community coordinators to help us make an initial assessment based on our selection criteria. The community coordinators interact with the community, so they are more familiar with the residents. The community coordinators helped us contact the potentially eligible subjects and briefly introduced our survey as an academic study concerning individual and household characteristics. The purpose of using community coordinators was also to increase the subjects’ trust in our study. Some subjects refused the survey invitation, but unfortunately, we do not know the refusal rate since community coordinators had not been instructed to keep exact track of number of refusals vs. acceptances. Note that the subjects that accepted the invitation were balanced on both birth year from 1976 to 1986 and gender between male and female in each of three sample cities, which thus suggests there is no obvious initial selection problem.

We then contacted the potentially eligible subjects by phone to make sure they met the requirements for our study. In the phone call, we introduced ourselves as academic researchers, referred to the contact they had had with the community coordinator who had identified them as eligible, and asked three questions to confirm they met our eligibility criteria. We asked the questions in a neutral way to avoid revealing that the study focused on OCP. For example, the age question asked in what year the subject was born rather than whether the subject was born between 1976 and 1986. If they were eligible, we then asked them whether they were interested in participating in our survey and told them the survey would take about one and a half hours. They were also told they would receive a show-up fee of 50 yuan and that they could earn additional money during the study; at the time of the experiment, 10 yuan = 1.6 USD. In addition, the subjects were clearly told that participation in the study was completely voluntary and that they could leave the experiment whenever they liked. In addition, they were told that all the survey information would be kept confidential and was only for the scientific research in question. If they verbally agreed, we then scheduled a survey appointment with them. This means that we obtained the subjects’ verbal consent before they participated in our experiments. When subjects arrived at the survey venue, we checked their identity to ensure that they had been invited and that the three study participation criteria were met. The experimenter then introduced the study and explained key rules, such as that the show-up fee would be paid only upon completion of the experiments and the questionnaire. The interviews and the experiments were conducted one-to-one. Our survey did not include any questions that are considered sensitive.

The study was organized as follows: we first conducted the five experiments, then subjects answered a questionnaire, and finally, we paid the subjects. The experiments were always conducted in the same order, since we wanted to reduce the risk of confusion by the experiments, and with five experiments there are 120 different ways the experiments could have been ordered. No information about the outcome or any other type of feedback was given between experiments. After the experiments, the subjects answered questionnaires about their socioeconomic background. The average duration of conducting the experiments and completing the questionnaire was about 1.5 hours. Finally, the subjects were paid the 50 yuan show-up fee, as well as any earnings from the risk and time preference experiments (if the subject had chosen payment today). The payments for the time preference experiment if a subject had chosen payment later were made via transfer to the subject’s bank account on the specified date. For the three other experiments, i.e., the competition, public good, and ultimatum games, subjects were invited to come back at a specific date for payment, since those payments partly depended on the decisions of others. For specific details on how the payoff decision was made in each experiment, see S1 Text. Moreover, for all the experiments, subjects were also asked a comprehension question to ensure that they understood how their total payoffs would be determined. Those who did not answer the question correctly received additional explanations to ensure they had understood.

### 3.3. Inclusivity in global research

Additional information regarding the ethical, cultural, and scientific considerations specific to inclusivity in global research is included in the Supporting Information (S2 Text).

## 4. Description of sample

### 4.1. Descriptive statistics

A total of 856 subjects participated in the experiments. In the post-experiment survey, we noticed that one subject in Guilin and one in Lanzhou had an invalid birth year, and hence we dropped them from the analyses. We also discovered that 72 subjects in the city of Guilin were in fact not born as citizens of Guilin. This was because they met the criterion of being born in what is now part of Guilin city, but the area in which they were born was not part of the city at the time they were born. Thus, to fulfil the strict criterion of implementation of OCP, we decided to drop these 72 observations. This leaves us with 782 subjects: 335 in Guilin, 200 in Wuxi, and 247 in Lanzhou. Table 2 presents descriptive statistics of the whole sample and for the three cities separately.

**Table 2:** Descriptive statistics.

Variable	Description	Whole sample	Guilin	Wuxi	Lanzhou	H_0_: No difference between cities (p-value)
*Individual control variables*						
Female	= 1 if female subject	0.49	0.49	0.50	0.50	0.94
		(0.50)	(0.50)	(0.50)	(0.50)	
Have children	= 1 if subject has at least one child	0.65	0.55	0.81	0.65	<0.01
		(0.48)	(0.50)	(0.39)	(0.48)	
Number children	No. of children if subject has children	1.04	1.04	1.02	1.04	0.64
		(0.19)	(0.20)	(0.16)	(0.19)	
Married	= 1 if married	0.78	0.70	0.89	0.76	<0.01
		(0.42)	(0.46)	(0.32)	(0.42)	
Only-child	= 1 if no siblings	0.73	0.71	0.82	0.70	0.01
		(0.44)	(0.45)	(0.39)	(0.46)	
Number siblings	No. of siblings if subject has siblings	1.25	1.14	1.32	1.34	0.02
		(0.44)	(0.41)	(0.48)	(0.71)	
Income	Own annual income in 10,000 yuan	4.83	3.77	6.51	4.89	<0.01
		(5.42)	(4.27)	(4.40)	(7.00)	
Household income	Annual household income in 10,000 yuan	13.0	10.38	18.52	12.05	<0.01
		(10.70)	(7.99)	(11.09)	(11.82)	
University	= 1 if university education	0.51	0.42	0.58	0.58	<0.01
		(0.38)	(0.41)	(0.35)	(0.36)	
*Parental control variables*						
Parents University	= 1 if at least one of the parents has university education	0.25	0.22	0.21	0.32	<0.01
		(0.43)	(0.41)	(0.40)	(0.47)	
No. of siblings, father	No. of siblings subject’s father has	3.61	3.60	3.49	3.71	0.29
		(1.76)	(1.90)	(1.63)	(1.65)	
No. of siblings, mother	No. of siblings subject’s mother has	3.82	3.89	3.70	3.81	0.31
		(1.71)	(1.78)	(1.68)	(1.65)	
High relative income	Higher relative income during subject’s childhood	0.27	0.31	0.28	0.21	0.03
		(0.44)	(0.46)	(0.45)	(0.41)	
Low relative income	Lower relative income during subject’s childhood	0.11	0.13	0.06	0.12	0.04
		(0.31)	(0.33)	(0.24)	(0.33)	
Number of individuals		782	335	200	247	

*Note*: We use a chi-square test for binary variables and a Kruskal-Wallis test for continuous data. Standard deviations in parentheses.

Women make up half of the sample, since this was a sampling criterion, and a large proportion of subjects are married. Of the 782 subjects, 73% are only-children, and among those who have siblings, the average number of siblings is 1.25. Out of the 65% who have children themselves, an overwhelming majority have only one child. The average yearly income is 48,300 yuan, and 51% have a university degree. Around 25% have at least one parent with university education, and the parents have on average several siblings and are therefore not generally only-children. Finally, about 27% of the subjects reported that their family income during their childhood (before age 16) was higher than average, while 11% reported a below average family income. If we compare the statistics across the three cities, we observe some differences. In Wuxi, both individual and household incomes are considerably higher, and a larger proportion of subjects grew up as only-children, are married, and have children themselves. The number of siblings is considerably higher in Lanzhou. As discussed in Carlsson et al. [33], by and large the sample is representative in terms of education level and gender compared with the total population of the three cities. By using the NBS [34], we identify a comparable city population (age between 25 and 39 and born in the main districts of the sample cities), and compare with our sample. The proportion of females is 49% in both the comparable city population and our sample. The proportion of males with a university degree is about 53% in both the comparable city population and our sample, while the proportion of females with a university degree is a bit higher in our sample (42% vs. 49%).

### 4.2. Distribution of birth years and the implementation of the OCP

Table 3 reports the distribution of subjects across birth years for the whole sample and for each of the three cities separately. We can see that the distribution of our sample subjects is quite balanced birth year-wise.

**Table 3:** Distribution of subjects across birth years.

Birth year	Whole sample	Guilin	Wuxi	Lanzhou
1976	9.6%	8.4%	13.0%	8.5%
1977	9.3%	9.3%	10.5%	8.5%
1978	11.0%	11.3%	10.5%	10.9%
1979	7.4%	9.0%	6.5%	6.1%
1980	5.9%	6.0%	7.0%	4.9%
1981	8.1%	7.2%	8.5%	8.9%
1982	10.4%	10.5%	9.0%	11.3%
1983	10.5%	9.9%	9.5%	12.2%
1984	10.1%	9.9%	9.0%	11.3%
1985	8.1%	8.4%	8.0%	7.7%
1986	9.7%	10.5%	8.5%	9.7%
Number of individuals	782	335	200	247

Note that in the early days of the OCP, instead of forbidding couples to have more than one child, it was strongly recommended that they have only one child. Therefore, we define the start of the “one is best, two at most” recommendation as the first stage of the OCP in this study. People born in July–September 1979 and onward, but before the policy became stricter, are classified as belonging to the first stage of the OCP. Clearly, we could think of other cutoff dates, and we investigate other cutoffs in a sensitivity analysis (S1 and S2 Tables). We define the second stage, with a stricter implementation of the OCP, as when a financial penalty in terms of a salary cut was imposed on couples with more than one child. The policy was made stricter at varying times in the three cities: In Guilin, subjects born from May 1981 and onward are defined as born under a stricter OCP. In Wuxi and Lanzhou, the corresponding cutoffs are June 1982 and April 1982, respectively. Table 4 presents the resulting distribution of subjects and the cutoff dates for the first and second stages of the OCP.

**Table 4:** Distribution of subjects based on implementation of the OCP in the three locations.

	Whole sample	Guilin	Wuxi	Lanzhou
	(n = 782)	(n = 335)	(n = 200)	(n = 247)
Before OCP	34.1%	34.0%	37.0%	32.0%
First stage OCP	16.5%	11.4%	23.0%	18.2%
Second stage OCP	49.4%	54.6%	40.0%	49.8%
Cutoff dates				
First stage OCP		20 Sept. 1979	31 July 1979	14 July 1979
Second stage OCP		18 May 1981	1 June 1982	20 April 1982

Table 5 presents descriptive statistics for the subjects, separated by the different stages of the OCP. The differences among the three groups are as expected. A larger share of those born before the OCP has siblings. Furthermore, more of them also have own children and are married, which is as expected, since these subjects are older.

**Table 5:** Descriptive statistics for subjects born before the OCP, during the first stage, and during the second stage.

	Before OCP	First stage OCP	Second stage OCP	H_0_: No difference between stages of the OCP
Female	0.51	0.55	0.46	0.16
	(0.51)	(0.50)	(0.50)	
Have children	0.84	0.73	0.49	<0.01
	(0.36)	(0.45)	(0.50)	
Number of children	1.04	1.03	1.03	0.61
	(0.21)	(0.18)	(0.16)	
Married	0.91	0.82	0.66	<0.01
	(0.29)	(0.40)	(0.48)	
Only-child	0.47	0.81	0.89	<0.01
	(0.50)	(0.39)	(0.31)	
Number of siblings (if any)	1.31	1.17	1.07	0.01
	(0.60)	(0.48)	(0.34)	
Income	4.88	5.20	4.66	0.24
	(5.57)	(4.22)	(5.67)	
Household income	12.49	14.05	12.98	0.08
	(10.50)	(9.90)	(11.02)	
University	0.38	0.54	0.60	<0.01
	(0.49)	(0.50)	(0.49)	
Number of individuals	267	129	386	

*Note*: We use a chi-square test for binary variables and a Kruskal-Wallis test for continuous data. Standard deviations in parentheses.

To establish that the OCP and the increased strictness of the policy did affect the household composition, we estimate models explaining the likelihood of being an only-child (binary probit) and the number of siblings (ordinary least squares), respectively. As independent variables, we use two indicator variables for the two stages of the OCP and controls for age, gender, and location fixed effects. We also added control variables for several family background variables that could have affected parents’ decision to have only one child or several children, such as parents’ number of siblings and education level and a variable capturing relative income level during subject’s childhood (before age 16). The two indicator variables for the policies are not perfectly correlated with age since we are using the variation in implementation dates among the three cities. Table 6 presents results of the two models.

**Table 6:** Regression models: The OCP and the likelihood of being an only-child and the number of siblings. Marginal effects for the probit model.

	Only-child	No. of siblings
	(probit)	(OLS)
First stage OCP	0.170***	-0.338***
	(0.038)	(0.076)
Second stage OCP	0.231***	-0.285**
	(0.087)	(0.111)
Age	Yes	Yes
Location	Yes	Yes
Gender	Yes	Yes
Parental control variables	Yes	Yes
Number of individuals	782	782

*Note*: OLS regression and clustered at individual level. Standard errors in parentheses.

*** significant at the 1% level,

** significant at the 5% level,

* significant at the 10% level.

The results of the probit regression show that the likelihood of being an only-child is considerably higher for the subjects born during the first or second stage of the OCP. More precisely, the likelihood is 17 and 23 percentage points higher compared with the period before the OCP. Similarly, according to the OLS model, the number of siblings is significantly lower for those born during the OCP than for those born before the policy. There are no differences between the two stages for only-child or number of siblings.

## 5. Results

### 5.1. The effect of the OCP: Descriptive results

Results from the risk and time preference experiments and the three experiments in the social domain are presented in Table 7. For the risk and uncertainty experiments, we report the ratio between the certainty equivalent at the switching point and the expected value for the lottery. We do this for a 50% probability of winning only; the results for a 10% and 90% probability of winning are similar to the ones for the 50% probability, results are available upon request. A ratio between the certainty equivalent and the expected value greater than one indicates that the subject is risk loving, while a ratio smaller than one indicates that the subject is risk averse. For the time preference experiment, we report estimates of the discount factor (*δ*) and present bias parameter (*β*) based on the regression model presented in the next section in Table 9. For the three behavioral experiments–the public good, competition, and ultimatum game–we report the average values of the observed behavior. For all experiments, we report the results separately for before the OCP, the first stage of the OCP, and the second stage of the OCP. In the lower panel of Table 7, we show the pairwise statistical tests across the different OCP stages.

**Table 7:** Descriptive statistics of main variables in the experiments.

	Risk	Uncertainty	Time		Public Good	Competition		Ultimatum	
	Certainty equivalent	Certainty equivalent	Discount factor	Present bias	Contribution (tokens)	Performance increase	Choose tournament	Offer	Min. accept. offer
Before OCP	0.90	0.65	0.976	0.998	8.06	0.64	0.28	19.8	14.9
	(0.30)	(0.34)	(0.001)	(0.010)	(4.39)	(2.38)	(0.45)	(2.06)	(5.63)
First stage OCP	0.99	0.75	0.977	0.986	8.22	0.53	0.36	19.6	15.2
	(0.33)	(0.34)	(0.001)	(0.007)	(4.19)	(2.32)	(0.48)	(2.14)	(6.05)
Second stage OCP	0.98	0.73	0.976	0.982	8.10	0.66	0.32	19.5	15.4
	(0.34)	(0.37)	(0.001)	(0.008)	(4.86)	(2.13)	(0.47)	(2.14)	(5.61)
No. individuals	782	782	782	782	782	782	782	782	782
*Note*: Standard deviations in parentheses.									
H_0_: No difference between OCP stages. P-values									
Before vs First	0.007	0.008	0.595	0.257	0.415	0.661	0.108	0.934	0.238
	(0.016)	(0.016)	(0.714)	(0.497)	(0.623)	(0.793)	(0.648)	(0.943)	(0.357)
Before vs Second	0.005	0.018	0.132	0.331	0.712	0.931	0.353	0.059	0.107
	(0.016)	(0.027)	(0.396)	(0.497)	(0.712)	(0.931)	(0.706)	(0.308)	(0.308)
First vs Second	0.601	0.350	0.088	0.745	0.269	0.610	0.339	0.154	0.943
	(0.601)	(0.420)	(0.396)	(0.745)	(0.623)	(0.793)	(0.706)	(0.308)	(0.943)

*Note*: The Benjamini and Hochberg adjusted p-values are shown in parentheses.

Subjects are, on average, slightly risk averse. If we compare ratios between the experiments with risky and uncertain outcomes, we find a higher tolerance for risky outcomes than for uncertain outcomes; that is, subjects are on average uncertainty averse. These results are in line with previous findings in the literature [35–37]. In the lower panel of Table 7, we show the pairwise statistical tests (Kruskal-Wallis) of the ratios for different OCP stages. We find some small differences in attitudes to risk and uncertainty among the three different subject groups separated by the different OCP stages. Overall, individuals born before the introduction of the OCP are more risk and uncertainty averse than those born after. We use the Benjamini and Hochberg correction to consider the fact that we make multiple comparisons [38], which Benjamini and Yekuteli recommend for most empirical settings. In total, we make six comparisons, three for each risk and uncertainty experiment, respectively [39]. Also, after the Benjamini and Hochberg correction, the results remain statistically significant at the 10% level (Table 7 shows the Benjamini and Hochberg adjusted p-values in parentheses for all the comparisons). Thus, there is an indication of subjects born after the introduction of the OCP being less risk and uncertainty averse.

There is support for the presence of present bias since the parameter for present bias (*β*) is statistically significantly different from one for the later stages of the OCP. However, there are no statistically significant differences between the stages of the OCP. At the same time, there is a statistically significant discount effect of future payments (*δ*), since for all stages the discount factor is statistically significantly different from one.

The average unconditional contribution to the public good is about the same across the three OCP stages, and Mann-Whitney tests confirm that there are no statistically significant differences across the three stages. The maximum contribution was 20 tokens, meaning that the average contribution share is around 40%, which is within the range of what is typically found in this type of experiment [e.g., 40–42]. By using the responses to the conditional contribution table, subjects can be classified into contributor types such as free riders and conditional cooperators [41]. Using a chi-square test, we investigate whether the distribution of contributor types is the same in all three stages, and we find that we cannot reject this hypothesis (p-value = 0.894).

For the experiment on competitiveness, we focus on two measures. First, we look at the change in performance going from Task 1, piece rate, to Task 2, tournament. This is a measure of competitiveness at the intensive margin. Second, we look at the share of subjects choosing tournament when faced with a choice between piece rate and tournament. There is no clear pattern of the differences in performance improvement among the three different stages of the OCP, and there are no statistically significant differences. The share of subjects choosing tournament is around 30%, and it is somewhat higher for subjects born after the introduction of the OCP. The share of subjects choosing tournament is considerably lower than what most other studies have found, including the original study by Niederle and Vesterlund [31, 43]. By using proportion tests, we find no statistically significant differences among the three different stages.

In the ultimatum bargaining game, subjects played both roles, and we thus report both the amount offered and the minimum acceptable offer for all subjects. The amount offered is slightly below 50% of the endowment, and the average offer is about the same across the three stages. An offer of 50% is in line with previous experiments [e.g., 44, 45]. The averages across the three stages are similar in economic terms. Using a Mann-Whitney test, we find one statistically significant difference at the 10% level in the amount offered: between those born pre-OCP and during the second stage of the OCP, but the significance does not hold for the Benjamin and Hochberg correction. The minimum acceptable offer is around 38% of the endowment, which is also largely in line with previous findings [46]. The minimum acceptable offer is again similar in all three stages of the OCP, and there are no statistically significant differences among the three stages using a Mann-Whitney test.

### 5.2. The effect of the OCP and of being an only-child: Regression models

The next step is to investigate differences in preferences across the three stages while controlling for the location and the age of the subject, but also including a set of individual and parental characteristics. The risk and uncertainty preferences are analyzed with an OLS regression, where the dependent variable is the ratio between the certainty equivalent at the switching point and the expected value for the lottery. The likelihood of choosing the tournament in the competition experiment is analyzed with a binary probit model, while all the other models are analyzed using an OLS regression. Results are reported in Table 8.

**Table 8:** Regression models of behavioral experiments with additional covariates.

	Risk	Uncertainty	Public Good	Competition		Ultimatum	
	Certainty equivalent	Certainty equivalent	Contribution	Performance increase	Choose tournament	Offer	Min. acceptable offer
First stage OCP	0.095**	0.136***	-0.190	-0.051	0.065	0.063	0.460
	(0.045)	(0.048)	(0.638)	(0.312)	(0.068)	(0.293)	(0.780)
Second stage OCP	0.076	0.170**	-0.756	0.199	-0.041	0.213	1.109
	(0.066)	(0.071)	(0.942)	(0.461)	(0.096)	(0.433)	(1.151)
Female	-0.081***	-0.075***	-0.863***	0.341**	-0.224***	0.033	-0.162
	(0.023)	(0.025)	(0.333)	(0.163)	(0.033)	(0.153)	(0.407)
Have children	-0.022	-0.065*	-0.515	0.432*	0.041	0.317	-0.187
	(0.036)	(0.037)	(0.486)	(0.237)	(0.048)	(0.223)	(0.593)
Married	0.003	-0.016	-0.007	-0.411	0.064	0.164	0.652
	(0.036)	(0.039)	(0.513)	(0.251)	(0.049)	(0.235)	(0.627)
Household income	-0.0004	0.002*	-0.004	-0.011	0.004**	0.009	-0.023
	(0.001)	(0.001)	(0.017)	(0.008)	(0.002)	(0.008)	(0.021)
University	-0.059**	-0.120***	-0.714**	0.052	0.030	-0.182	-0.546
	(0.024)	(0.026)	(0.349)	(0.171)	(0.036)	(0.160)	(0.426)
Parent university	-0.028	-0.009	-0.160	-0.175	0.053	-0.161	-0.569
	(0.028)	(0.030)	(0.399)	(0.195)	(0.042)	(0.183)	(0.488)
No. of siblings, father	-0.001	-0.006	0.004	-0.013	0.005	0.013	-0.089
	(0.007)	(0.007)	(0.094)	(0.046)	(0.010)	(0.043)	(0.115)
No. of siblings, mother	0.014**	0.005	-0.042	0.069	0.017*	0.081*	-0.028
	(0.007)	(0.007)	(0.097)	(0.048)	(0.010)	(0.045)	(0.119)
High relative income	0.028	-0.002	-0.402	-0.177	0.021	0.138	0.387
	(0.027)	(0.029)	(0.3869	(0.189)	(0.040)	(0.177)	(0.471)
Low relative income	0.075*	0.104**	-0.415	0.233	0.008	-0.097	-0.739
	(0.039)	(0.042)	(0.554)	(0.271)	(0.057)	(0.254)	(677)
Age	Yes	Yes	Yes	Yes	Yes	Yes	Yes
Location	Yes	Yes	Yes	Yes	Yes	Yes	Yes
Number of individuals	781	781	781	781	781	781	781

*Note*: Standard errors in parentheses.

*** significant at the 1% level,

** significant at the 5% level,

* significant at the 10% level.

Inclusion of control variables for individual and parental characteristics and location do not affect the main results. There are not any statistically significant differences in the public good, competition, or ultimatum games across the three different stages. There are still statistically significant differences among the different stages of the OCP with respect to risk and uncertainty preferences. We find that female subjects are more uncertainty averse, contribute less to the public good, and less likely to choose the tournament. However, females increase their performance significantly more than men under competition. In addition, subjects with a university degree are more risk averse and contribute less to the public good. There are no statistically significant differences in preferences due to parents’ education or household income, with two exceptions: Those with higher incomes are less uncertainty averse and more likely to choose tournament in the competition experiment. Those who experience a lower than an average income level during childhood (before age 16) are both more risk taking and less uncertainty averse than others.

The design of the time preference experiment allows us to estimate the beta-delta model by Laibson [47]. This is an exponential discounting model (*δ*) allowing for a preference to receive a payment immediately (present bias), while any future event is given a lower value (*β*). We estimate the following regression model, based on Burks et al. [48], which assumes an additive error term

$$\text{ln}\left(x\right)-\text{ln}\left(y\right)=\text{ln}\left(\beta \right)t_{0}+\text{ln}\left(\delta \right)\left(t_{later}-t_{sooner}\right)+\varepsilon ,$$ (1)

where *x* is the sooner payment amount, *y* is the later payment amount, *t*_0_ is a dummy variable equal to one if the sooner payment is today, *β* is the present bias parameter, *δ* is the discount parameter, and *ε* is the error term. In Table 9, we report the regression results.

**Table 9:** Regression model of time preferences.

	Model 1		Model 2
log(*β*)	0.001		-0.003
	(0.004)		(0.005)
log(*δ*)	-0.009***		-0.009***
	(0.0005)		(0.0007)
log(*β*) × First stage OCP	-0.005		-0.005
	(0.005)		(0.005)
log(*δ*) × First stage OCP	0.0003		0.0002
	(0.001)		(0.0006)
log(*β*) × Second stage OCP	-0.007		-0.006
	(0.007)		(0.007)
log(*δ*) × Second stage OCP	0.002		0.001
	(0.001)		(0.001)
log(*β*) × Female			0.0006
			(0.003)
log(*δ*) × Female			-0.0002
			(0.0003)
log(*β*) × Have children			-0.0004
			(0.003)
log(*δ*) × Have children			0.0004
			(0.0005)
log(*β*) × Married			0.0003
			(0.004)
log(*δ*) × Married			-0.0004
			(0.0005)
log(*β*) × Household income			0.00005
			(0.0001)
log(*δ*) × Household income			-0.00001
			(0.00001)
log(*β*) × University			-0.0029
			(0.0029)
log(*δ*) × University			0.001**
			(0.0003)
log(*β*) × Parent university			0.002
			(0.003)
log(*δ*) × Parent university			-0.0002
			(0.0004)
log(*β*) × No. of siblings, mother			0.0005
			(0.0007)
log(*δ*) × No. of siblings, mother			-0.0001
			(0.0001)
log(*β*) × No. of siblings, father			0.0007
			(0.0008)
log(*δ*) × No. of siblings, father			-0.0001
			(0.0001)
log(*β*) × High relative income			0.001
			(0.003)
log(*δ*) × High relative income			-0.0006*
			(0.0004)
log(*β*) × Low relative income			-0.006
			(0.004)
log(*δ*) × Low relative income			0.001**
			(0.0006)
Age	Yes		Yes
Location fixed effects	Yes		Yes
Number of observations	3128		3128
Number of individuals	782		782
H_0_: *β* = 1; H_0_: *δ* = 0	*β* ^*a*^	*δ* ^*a*^	
Before OCP	0.998	0.976***	
	(0.010)	(0.001)	
First stage OCP	0.986**	0.977***	
	(0.007)	(0.001)	
Second stage OCP	0.982**	0.980***	
	(0.008)	(0.001)	
H_0_: No difference between OCP stages	P-values		
Before vs First	0.257	0.595	
Before vs Second	0.331	0.132	
First vs Second	0.745	0.088	

*Note*: OLS regression and clustered at individual level. Standard errors in parentheses.

*** significant at the 1% level,

** significant at the 5% level, and

* significant at the 10% level.

As we have seen, there is support for the presence of present bias, since the *β*-parameter is statistically significantly different from one for the later stages of the OCP. However, there are no statistically significant differences between the stages of the OCP. Furthermore, there is a statistically significant difference at the 10% level in impatience (*δ*) between subjects born during the first and second stages of the OCP. However, the differences are small in economic terms. Including additional control variables, we only find that subjects with a university degree are more impatient.

### 5.3. Heterogeneous effects of the OCP

It is possible that the OCP has affected different subgroups of our sample differently. The most obvious candidate is gender differences in preferences and behavior [49]. Traditionally, women have been discriminated against in China [50, 51]. During the OCP, the preference for boys increased [52] and the “little emperor syndrome” is seen as potentially stronger among boys in China [53]. On the other hand, girls in one-child families could benefit from being an only-child since families may invest more in them when there is no competition from siblings and especially not from brothers [26]. Thus, a consequence of the OCP, with an increased and substantial sex-ratio difference after its introduction, is that it might have affected preferences among men and women differently. We also know from previous experiments that there might be differences in preferences between men and women. For example, some studies find that women are more risk averse than men [e.g., 49], although this is far from a general finding [54]. We therefore conduct the same analyses as in Sections 5.1 and 5.2, but separately for men and women (S3 and S4 Tables). These analyses do not reveal any important gender differences in preferences and behavior. Overall, there are only a small number of statistically significant differences across the stages of the OCP when we conduct the analyses separately for men and women. However, the fact that those born after the introduction of OCP are less averse to uncertainty (see Table 8) is driven by males; males born during the OCP stages differ significantly from males born pre-OCP, but no such differences are found for women.

The three cities differ in many dimensions, including in the specific rules of the OCP. We therefore estimate separate models for each city (S5 and S6 Tables). Overall, we find only a few differences among subjects born in the three cities, but in Wuxi, those born after the introduction of the OCP were less risk and uncertainty averse than those born pre-OCP. In the behavioral experiments, those born after the OCP offered significantly more in the ultimatum game also in Wuxi.

### 5.4. The effects of being the only-child: IV-regressions

In Table 10 we use an IV regression, where being born during the first or second OCP stage is used as an instrument for being an only-child. We include location fixed effects, age of the subject, and the same set of individual and parental characteristics as in previous analyses. For all models, we only report the estimate of the coefficient for only-child; full results are available upon request.

**Table 10:** IV regression models of behavioral experiments where we used being born under the first or second OCP stage as an instrument for being an only-child.

	Risk	Uncertainty	Time		Public Good	Competition		Ultimatum	
	Certainty equivalent	Certainty equivalent	Discount factor	Present bias	Contribution	Performance increase	Choose tournament	Offer	Min. acceptable offer
Only-child	0.385**	0.538**	0.0006	-0.020	-0.617	-0.267	0.855	0.213	1.676
	(0.195)	(0.224)	(0.003)	(0.0196351)	(2.527)	(1.235)	(0.653)	(1.161)	(3.090)
Age	Yes	Yes	Yes		Yes	Yes	Yes	Yes	Yes
Location dummies	Yes	Yes	Yes		Yes	Yes	Yes	Yes	Yes
Individual and parental controls	Yes	Yes	Yes		Yes	Yes	Yes	Yes	Yes
Number of individuals	781	781	782		781	781	781	781	781

*Note*: Standard errors in parentheses.

*** Significant at the 1% level,

** significant at the 5% level,

* significant at the 10% level.

The results are, as expected, in line with the previous analyses. We find a statistically significant effect of being an only-child on risk and uncertainty preferences, but no other statistically significant effects.

### 5.5. Robustness checks

#### 5.5.1. Using alternative cutoff dates and considering only firstborns

Since a pregnancy takes nine months, firstborns born close to any of the dates when the local authorities implemented the OCP are more likely to be an only-child. The gap between the birthdate and implementation of the two OCP stages is simply too tight for these children to have siblings. As a robustness check, we change the cutoff dates to one year earlier than the dates used in the main analysis. In Table 11, we show the distribution of the subjects according to our main and alternative cutoff dates.

**Table 11:** Distribution of subjects based on implementation of the OCP with both cutoff dates in the three locations.

	Guilin		Wuxi		Lanzhou	
Cutoff definitions	Main	Alt.	Main	Alt.	Main	Alt.
Cutoff between before OCP and second stage OCP	20 Sept. 1979	20 Sept. 1978	31 July 1979	31 July 1978	14 July 1979	14 July 1978
Cutoff between first and second stages OCP	18 May 1980	18 May 1979	1 June 1982	1 June 1981	20 April 1982	20 April 1981
Before OCP	34.0%	24.8%	37.0%	29.5%	32.0%	22.3%
First stage OCP	11.3%	15.5%	23.0%	21.0%	18.2%	17.8%
Second stage OCP	54.6%	59.7%	40.0%	49.5%	49.8%	59.9%

We investigate differences between the stages by using this alternative cutoff (S1 Table). With the new classification, the likelihood of being an only-child is 19 percentage points higher if a child was born during the first stage of the OCP policy, and it is 30 percentage points higher if they were born during the second stage (S1 Table). Overall, there are no significant differences compared with the main analysis, with a few exceptions: The effects of being born before the OCP on being less risk and uncertainty seeking are less prominent, and the results are now insignificant in most cases (S2 Table). Subjects born during the second stage of the OCP are more patient and more likely to choose tournament than those born before the OCP.

We also investigate the effects of the policy by considering only firstborns; firstborns born before the policy would have been only-children had they been born after OCP implementation (S7 Table). The results are very similar to the ones in the main analysis: We again find significant, but small, effects in risk and uncertainty preferences between those born before and after OCP implementation, while we do not find any corresponding differences in the other experiments.

#### 5.5.2. Investigating the effects of the 1999 university reform

Finally, we investigate the effects of the large university reform implemented in 1999 [55]. Although the central government’s education policy had increased the number of people with a college degree from 0.4 million to 1.08 million from 1978 to 1998, the expansion in 1999 resulted in an increase of newly admitted students by around 40% [55, 56]. People in China typically enroll in higher education at age 18 [55]. Hence, subjects born after 1980 have benefitted from this university reform. We conduct three robustness checks to investigate potential effects of this education reform on preferences and behavior. First, we use an indicator to identify post university-reform time in the regressions (S8 and S9 Tables). The dummy variable for the university reform is sometimes statistically significant, but there are no sizeable differences compared with the main analysis. Second, we control for university education and estimate separate models for those with and without a university degree (S10–S12 Tables). Third, using subjects born before the education reform, we estimate a model predicting the likelihood of getting a university degree. This model is then used to predict university education for the sample born after the education reform. We then perform the same analyses as in the main section based on these two subsamples instead (S10–S12 Tables).

Overall, there are no large differences compared with the main analysis. The main difference is that we find a statistically significant difference only in risk preferences between those born before and during the OCP for those with a university degree (see S10 Table). Thus, the university reform might have had an impact on the distribution of risk preferences among the sample, but not on behavior in the domains we have investigated experimentally.

## 6. Discussion

There is an extensive literature on and interest in the long-term consequences of being an only-child on behavior, especially when the trend around the world is toward fewer children per family. The empirical evidence on this topic is mixed [e.g., 1, 2]. One obvious challenge for any research in this area is the lack of any family policy that allows for a clean identification strategy. In this paper, we exploit the best-known family policy of all times, China’s one-child policy (OCP), implemented in 1979. The OCP led to an increased number of one-child families and an increased gender imbalance. An important question is whether the policy influences people’s preferences and behavior. Popular wisdom seems to be that being an only-child has a negative impact on various social skills. However, the overall conclusion from the literature indicates that the effects of being an only-child on preferences and behavior are not all that evident. Moreover, there is a lack of studies investigating the effects of the OCP on preferences and behavior among adults.

We find very little support for effects of the policy on preferences in the social domain or on time preferences. We find some statistically significant, while small in magnitude, differences when it comes to risk and uncertainty preferences, where the latter is explained by males born after the introduction of the OCP. Moreover, our robustness analyses suggest that part of the effect on risk preferences could be due to the university reform, but not the OCP. Even when including cities that differ in timing of intensifying the policy, location, population size, and GDP per capita, we are not able to identify any effects of the OCP on preferences.

It is interesting to contrast our results with those of Cameron et al. [19], a study that, similar to ours, includes a set of experiments and uses a sample of subjects born before and after the OCP was introduced in 1979. Our study complements and extends their study. We conducted our experiments in three different cities, which allows us to explore differences in timing in introducing the first and second stages of the OCP. Moreover, our sampling frame required that subjects were born and still lived in the same place, and that they were Han Chinese. Since both their study and ours include risk and competition experiments, we can make a direct comparison for these experiments. They found that subjects born after 1979 were significantly more risk averse than those born before, whereas we found some evidence for the opposite. We found no statistically significant differences in tournament entry decisions among the OCP stages, whereas they found a weakly significant difference in the likelihood to participate in a tournament between subjects born before and after the introduction of the OCP. Thus, as opposed to Cameron et al. [19], we do not find any convincing evidence that the OCP has affected preferences or created “little emperors.”

Using the introduction and timing of the OCP as instruments, we are able to investigate the effects of being an only-child on preferences. The results are, as expected, in line with the previous findings on the direct effects of the OCP. Thus, we contribute to a vast literature that has reported mixed results on preferences and behavior of being an only-child. In summary, we do not find any significant impact of the OCP among adults on uncertainty and time preferences or on various preferences in the social domain. We conclude that the negative stereotype of being a little emperor is just a stereotype. Mõttus et al. demonstrate that there exists a general negative stereotype of being an only-child among both only-children themselves and children with siblings [54]. According to Mõttus et al., stereotypes are however not meaningless [57]. They are instead social constructions affecting people’s behavior. For example, if only-children are believed to have poorer social skills, it can motivate families to have more children, and can also motivate the parents to pay more attention to strengthening the social skills of their only-child.

## Supporting information

S1 Table Robustness checks with new cutoff dates (minus one year). Regression models: The OCP and the likelihood of being an only-child and the number of siblings.
(DOCX)

S2 Table Descriptive statistics of main variables in the experiments with alternative cutoff date. (DOCX)

S3 Table Regression models of risk and behavioral experiments by gender. (DOCX)

S4 Table Regression model of time preference by gender. (DOCX)

S5 Table Regression models of risk and behavioral experiments by location. (DOCX)

S6 Table Regression models of time preference by location. (DOCX)

S7 Table Descriptive statistics of main variables in the experiments with only firstborns. (DOCX)

S8 Table Regression models of risk and behavioral experiments with university reform as explanatory variable. (DOCX)

S9 Table Regression model of time preferences, university reform as explanatory variable. (DOCX)

S10 Table Regression model of risk and uncertainty preferences to test effects of university reform. (DOCX)

S11 Table Regression model of time preferences to test effects of university reform. (DOCX)

S12 Table Regression models of behavioral experiments to test effects of university reform. (DOCX)

S1 Text Description of experiments. (DOCX)

S2 Text Inclusivity in global research. (DOCX)
