# Supplementary material for: Are only-children different? Evidence from a lab-in-the-field experiment of the Chinese one-child policy
Source: PLoS One. 2022 Nov 8;17(11):e0277210. doi: 10.1371/journal.pone.0277210 (PMC9642884; doi:10.1371/journal.pone.0277210)
Supplement: S1 Table — Regression models: The OCP and the likelihood of being an only-child and the number of siblings. (DOCX) [file pone.0277210.s001.docx]

**S1 Table.** **Robustness checks with new cutoff dates (minus one year). Regression models: the OCP and the likelihood of being an only child and the number of siblings.**

|  | Only child | No. of siblings |
| --- | --- | --- |
| First stage OCP | 0.189^***^  (0.033) | -0.450^***^  (0.068) |
| Second stage OCP | 0.303^***^  (0.087) | -0.459^***^  (0.102) |
| Time | Yes | Yes |
| Location | Yes | Yes |
| Gender | Yes | Yes |
| Parental controls | Yes | Yes |
| Number of individuals | 782 | 782 |

*Note*: OLS regression and clustered at individual level. Standard errors in parentheses. *** significant at 1% level, ** significant at 5% level, * significant at 10% level.
