# Supplementary material for: Are only-children different? Evidence from a lab-in-the-field experiment of the Chinese one-child policy
Source: PLoS One. 2022 Nov 8;17(11):e0277210. doi: 10.1371/journal.pone.0277210 (PMC9642884; doi:10.1371/journal.pone.0277210)
Supplement: S2 Table — (DOCX) [file pone.0277210.s002.docx]

**S2 Table. Descriptive statistics of main variables in the experiments with alternative cutoff date**

|  | Risk | Uncertainty | Time | | Public Good | Competition | | Ultimatum | |  |
| --- | --- | --- | --- | --- | --- | --- | --- | --- | --- | --- |
|  |  |  | Discount factor | Present bias | Contribution  (tokens) | Performance increase | Choose tournament | Offer | Min. accept. offer |  |
| Before OCP | 0.92  (0.31) | 0.66  (0.34) | 0.976  (0.001) | 0.998  (0.010) | 8.04  (4.17) | 0.70  (2.33) | 0.26  (0.44) | 19.94  (1.76) | 15.04  (5.68) |  |
| First stage OCP | 0.94  (0.31) | 0.72  (0.37) | 0.976  (0.001) | 0.988  (0.007) | 8.07  (4.54) | 0.49  (2.51) | 0.33  (0.47) | 19.64  (2.24) | 14.53  (5.85) |  |
| Second stage OCP | 0.97  (0.34) | 0.72  (0.36) | 0.978  (0.001) | 0.984  (0.006) | 8.15  (4.79) | 0.64  (2.13) | 0.33  (0.47) | 19.45  (2.20) | 15.43  (5.64) |  |
| No. individ. | 782 | 782 | 782 | 782 | 782 | 782 | 782 | 782 | 782 |  |
| H_0_: No difference between OCP stages. P-values | | | | | | | | | | |
| Before vs First | 0.758 | 0.134 | 0.664 | 0.307 | 0.938 | 0.412 | 0.139 | 0.241 | 0.616 |  |
| Before vs Second | 0.129 | 0.053 | 0.091 | 0.402 | 0.819 | 0.800 | 0.060 | 0.012 | 0.252 |  |
| First vs Second | 0.318 | 0.938 | 0.009 | 0.770 | 0.925 | 0.485 | 1.000 | 0.418 | 0.104 |  |

*Note*: Standard deviations in parentheses.
