# Supplementary material for: Are only-children different? Evidence from a lab-in-the-field experiment of the Chinese one-child policy
Source: PLoS One. 2022 Nov 8;17(11):e0277210. doi: 10.1371/journal.pone.0277210 (PMC9642884; doi:10.1371/journal.pone.0277210)
Supplement: S3 Table — (DOCX) [file pone.0277210.s003.docx]

**S3 Table. Regression models of risk and behavioral experiments by gender**

|  | Public Good | | Comp perform | | Comp entry | | Ultimat. Offer | | Ultimat. accept | |
| --- | --- | --- | --- | --- | --- | --- | --- | --- | --- | --- |
|  | Men | Women | Men | Women | Men | Women | Men | Women | Men | Women |
| First stage OCP | -0.05  (0.98) | -0.50  (0.82) | -0.25  (0.44) | 0.30  (0.46) | 0.005  (0.10) | 0.13  (0.09) | 0.56  (0.54) | -0.37  (0.38) | 2.01*  (1.09) | -1.23  (1.13) |
| Second stage OCP | -1.17  (1.36) | -0.63  (1.26) | -0.07  (0.60) | 0.83  (0.70) | -0.01  (0.13) | -0.01  (0.13) | 0.81  (0.62) | -0.26  (0.59) | 2.07  (1.51) | -0.21  (1.74) |
| Age | Yes | Yes | Yes | Yes | Yes | Yes | Yes | Yes | Yes | Yes |
| Location | Yes | Yes | Yes | Yes | Yes | Yes | Yes | Yes | Yes | Yes |
| Parental controls | Yes | Yes | Yes | Yes | Yes | Yes | Yes | Yes | Yes | Yes |
| Number of individuals | 395 | 386 | 395 | 386 | 395 | 386 | 395 | 386 | 395 | 386 |
|  | Risk | | Uncertainty | |  |  |  |  |  |  |
|  | Men | Women | Men | Women |  |  |  |  |  |  |
| First stage OCP | 0.10  (0.06) | 0.06  (0.06) | 0.16^*^*  (0.07) | 0.08  (0.07) |  |  |  |  |  |  |
| Second stage OCP | 0.12  (0.09) | -0.02  (0.10) | 0.20^**^  (0.10) | 0.05  (0.11) |  |  |  |  |  |  |
| Number of individuals | 395 | 386 | 395 | 386 |  |  |  |  |  |  |

*Note*: Standard errors in parentheses. *** significant at 1% level, ** significant at 5% level, * significant at 10%
