# Supplementary material for: Are only-children different? Evidence from a lab-in-the-field experiment of the Chinese one-child policy
Source: PLoS One. 2022 Nov 8;17(11):e0277210. doi: 10.1371/journal.pone.0277210 (PMC9642884; doi:10.1371/journal.pone.0277210)
Supplement: S4 Table — (DOCX) [file pone.0277210.s004.docx]

**S4 Table. Regression model of time preference by gender**

|  | Men | Women |
| --- | --- | --- |
| $\log\left( \beta\right)$ | 0.003  (0.005) | -0.001  (0.005) |
| $\mathrm{Log} \left( \delta\right)$ | -0.009^***^  (0.001) | -0.009^***^  (0.001) |
| $\log\left( \beta\right)$ $\times$First stage OCP | -0.012^*^  (0.007) | 0.001  (0.007) |
| $\log\left( \delta\right)$ $\times$First stage OCP | -0.001  (0.001) | 0.001  (0.001) |
| $\log\left( \beta\right)$ $\times$Second stage OCP | -0.008  (0.009) | -0.004  (0.012) |
| $\log\left( \delta\right)$ $\times$Second stage OCP | 0.001  (0.001) | 0.002^*^  (0.001) |
| Age | Yes | Yes |
| Location | Yes | Yes |
| Number of observations | 1,584 | 1,544 |
| Number of individuals | 396 | 386 |

*Note*: OLS regression and clustered at individual level. Standard errors in parentheses. *** significant at 1% level, ** significant at 5% level, * significant at 10% level.
