# Supplementary material for: Are only-children different? Evidence from a lab-in-the-field experiment of the Chinese one-child policy
Source: PLoS One. 2022 Nov 8;17(11):e0277210. doi: 10.1371/journal.pone.0277210 (PMC9642884; doi:10.1371/journal.pone.0277210)
Supplement: S5 Table — (DOCX) [file pone.0277210.s005.docx]

**S5 Table. Regression models of risk and behavioral experiments by location**

|  | Risk | Uncertainty | Public Good | Competition | | Ultimatum | |
| --- | --- | --- | --- | --- | --- | --- | --- |
|  |  |  | Contribution | Performance increase | Choose tournament | Offer | Min. accept offer |
|  |  |  | Guilin (Number of individuals = 335) | | | | |
| First stage OCP | 0.031  (0.070) | 0.062  (0.077) | -0.658  (0.960) | 0.432  (0.454) | 0.176^*^  (0.106) | 0.033  (0.402) | -0.952  (1.195) |
| Second stage OCP | 0.082  (0.092) | 0.077  (0.101) | -0.640  (1.268) | 0.508  (0.599) | 0.090  (0.125) | 0.085  (0.531) | -0.903  (1.578) |
|  |  |  | Wuxi (Number of individuals = 200) | | | | |
| First stage OCP | 0.161^*^  (0.092) | 0.186^**^  (0.092) | -0.063  (1.336) | -0,707  (0.692) | -0.02  (0.115) | 1.167^**^  (0.573) | 1.353  (1.605) |
| Second stage OCP | 0.113  (0.151) | 0.238  (0.150) | -1.826  (2.187) | -0.690  (1.134) | 0.011  (0.197) | 2.493^***^  (0.938) | 3.821  (2.628) |
|  |  |  | Lanzhou (Number of individuals = 247) | | | | |
| First stage OCP | 0.067  (0.084) | 0.146  (0.096) | -1.240  (1.195) | -0.048  (0.585) | 0.024  (0.127) | -0.357  (0.628) | 2.276  (1.488) |
| Second stage OCP | 0.034  (0.012) | 0.201  (0.143) | -2.929*  (1.777) | 0.300  (0.871) | -0.174  (0.185) | -0.722  (0.934) | 3.023  (2.213) |

*Note*: Age fixed effects in all models, except Lanzhou. Standard errors in parentheses. *** significant at 1% level, ** significant at 5% level, * significant at 10% level.
