# Supplementary material for: Are only-children different? Evidence from a lab-in-the-field experiment of the Chinese one-child policy
Source: PLoS One. 2022 Nov 8;17(11):e0277210. doi: 10.1371/journal.pone.0277210 (PMC9642884; doi:10.1371/journal.pone.0277210)
Supplement: S6 Table — (DOCX) [file pone.0277210.s006.docx]

**S6 Table. Regression models of time preference by location**

|  | City | | |
| --- | --- | --- | --- |
|  | Guilin | Wuxi | Lanzhou |
| $\log\left( \beta\right)$ | 0.006  (0.005) | -0.011^*^  (0.006) | -0.009  (0.006) |
| $\log\left( \delta\right)$ | -0.009^***^  (0.001) | -0.004^***^  (0.009) | -0.010^***^  (0.001) |
| $\log\left( \beta\right)$ $\times$First stage OCP | -0.004  (0.008) | -0.004  (0.009) | -0.013  (0.008) |
| $\log\left( \delta\right)$ $\times$First stage OCP | -0.001  (0.001) | -0.0002  (0.001) | 0.002^*^  (0.001) |
| $\log\left( \beta\right)$ $\times$Second stage OCP | 0.001  (0.011) | -0.009  (0.016) | -0.020^*^  (0.012) |
| $\log\left( \delta\right)$ $\times$Second stage OCP | 0.002  (0.001) | 0.002  (0.002) | 0.002  (0.002) |
| Number of observations | 1,340 | 800 | 988 |
| Number of individuals | 335 | 200 | 247 |

*Note*: OLS regression and clustered at individual level. Standard errors in parentheses. *** significant at 1% level, ** significant at 5% level, * significant at 10% level.
