# Supplementary material for: Are only-children different? Evidence from a lab-in-the-field experiment of the Chinese one-child policy
Source: PLoS One. 2022 Nov 8;17(11):e0277210. doi: 10.1371/journal.pone.0277210 (PMC9642884; doi:10.1371/journal.pone.0277210)
Supplement: S7 Table — (DOCX) [file pone.0277210.s007.docx]

**S7 Table. Descriptive statistics of main variables in the experiments with only firstborns**

|  | Risk | Uncertainty | Time | | Public Good | Competition | | Ultimatum | |  |
| --- | --- | --- | --- | --- | --- | --- | --- | --- | --- | --- |
|  |  |  | Discount factor | Present bias | Contribution  (tokens) | Performance increase | Choose tournament | Offer | Min. accept. offer |  |
| Before OCP | 0.90  (0.29) | 0.62  (0.33) | 0.976  (0.002) | 0.996  (0.011) | 7.72  (4.23) | 0.67  (2.53) | 0.27  (0.45) | 19.87  (1.61) | 14.95  (5.70) |  |
| First stage OCP | 0.99  (0.34) | 0.74  (0.34) | 0.977  (0.001) | 0.989  (0.008) | 8.08  (4.31) | 0.47  (2.34) | 0.34  (0.48) | 19.65  (2.11) | 15.18  (5.83) |  |
| Second stage OCP | 0.97  (0.34) | 0.73  (0.37) | 0.980  (0.001) | 0.984  (0.009) | 8.02  (4.80) | 0.63  (2.13) | 0.31  (0.46) | 19.43  (2.18) | 15.48  (5.55) |  |
| No. individuals. | 637 | 637 | 637 | 637 | 637 | 637 | 637 | 637 | 637 |  |
| H_0_: No difference between OCP stages. P-values | | | | | | | | | | |
| Before vs First | 0.009 | 0.014 | 0.530 | 0.541 | 0.631 | 0.465 | 0.290 | 0.952 | 0.257 |  |
| Before vs Second | 0.005 | 0.017 | 0.208 | 0.495 | 0.583 | 0.938 | 0.430 | 0.041 | 0.068 |  |
| First vs Second | 0.620 | 0.375 | 0.197 | 0.699 | 0.211 | 0.699 | 0.295 | 0.120 | 0.907 |  |

*Note*: Standard deviations in parentheses.
