# Supplementary material for: Are only-children different? Evidence from a lab-in-the-field experiment of the Chinese one-child policy
Source: PLoS One. 2022 Nov 8;17(11):e0277210. doi: 10.1371/journal.pone.0277210 (PMC9642884; doi:10.1371/journal.pone.0277210)
Supplement: S8 Table — (DOCX) [file pone.0277210.s008.docx]

**S8 Table. Regression models of risk and behavioral experiments with university reform as explanatory variable**

|  | Risk | Uncertainty | Public Good | Competition | | Ultimatum | | |
| --- | --- | --- | --- | --- | --- | --- | --- | --- |
|  |  |  | Contribution | Performance increase | Choose tournament | Offer | | Min. acceptable offer |
| First stage OCP | 0.121^**^  (0.049) | 0.142^***^  (0.053) | -0.596  (0.692) | 0.099  (0.339) | 0.152  (0.197) | 0.232  (0.317) | | -0.174  (0.852) |
| Second stage OCP | 0.158^*^  (0.020) | 0.212^**^  (0.088) | -0.151  (1.113) | 0.478  (0.553) | -0.082  (0.320) | 0.793  (0.517) | | -0.247  (1.390) |
| University reform | -0.113^*^  (0.060) | -0.090  (0.066) | 0.749  (0.850) | -0.284  (0.416) | 0.113  (0.240) | -0.649*  (0.390) | | 1.688  (1.047) |
| Age | Yes | Yes | Yes | Yes | Yes | | Yes | Yes |
| Location | Yes | Yes | Yes | Yes | Yes | | Yes | Yes |
| Number of individuals | 782 | 782 | 782 | 782 | 782 | | 782 | 782 |
