# Supplementary material for: Are only-children different? Evidence from a lab-in-the-field experiment of the Chinese one-child policy
Source: PLoS One. 2022 Nov 8;17(11):e0277210. doi: 10.1371/journal.pone.0277210 (PMC9642884; doi:10.1371/journal.pone.0277210)
Supplement: S9 Table — (DOCX) [file pone.0277210.s009.docx]

**S9 Table. Regression model of time preferences, university reform as explanatory variable**

|  | Coefficient |
| --- | --- |
| $\log\left( \beta\right)$ | -0.002  (0.002) |
| $\log\left( \delta\right)$ | -0.010^***^  (0.000) |
| $\log\left( \beta\right)$ $\times$First stage OCP | -0.003  (0.005) |
| $\log\left( \delta\right)$ $\times$First stage OCP | 0.001  (0.001) |
| $\log\left( \beta\right)$ $\times$Second stage OCP | -0.000  (0.007) |
| $\log\left( \delta\right)$ $\times$Second stage OCP | -0.001  (0.001) |
| $\log\left( \beta\right) \times$ University reform | -0.004  (0.006) |
| $\log\left( \delta\right)$ $\times$ University reform | 0.001^*^  (0.001) |
| Age | Yes |
| Location | Yes |
| Number of observations | 3128 |
| Number of individuals | 782 |

*Note*: Clustered at individual level. Standard errors in parentheses. *** significant at 1% level, ** significant at 5% level, * significant at 10% level.
