# Supplementary material for: Are only-children different? Evidence from a lab-in-the-field experiment of the Chinese one-child policy
Source: PLoS One. 2022 Nov 8;17(11):e0277210. doi: 10.1371/journal.pone.0277210 (PMC9642884; doi:10.1371/journal.pone.0277210)
Supplement: S10 Table — (DOCX) [file pone.0277210.s010.docx]

**S10 Table. Regression model of risk and uncertainty preferences to test effects of university reform**

|  | **Using observed status of university education** | | | | **Using predicted status of university education** | | | |
| --- | --- | --- | --- | --- | --- | --- | --- | --- |
|  | Risk | | Uncertainty | | Risk | | Uncertainty | |
|  | No univ. | Univ. | No univ. | Univ. | No univ. | Univ. | No univ. | Univ. |
| First stage OCP | 0.103  (0.140) | 0.152^**^  (0.069) | 0.175^**^  (0.075) | 0.140^*^  (0.076) | 0.073  (0.070) | 0.174^***^  (0.068) | 0.098  (0.075) | 0.204  (0.075) |
| Second stage OCP | 0.177  (0.121) | 0.157  (0.107) | 0.299^**^  (0.129) | 0.158  (0.117) | 0.155  (0.113) | 0.144  (0.114) | 0.152  (0.122) | 0.280  (0.125) |
| University reform | -0.099  (0.091) | -0.130  (0.11) | -0.056  (0.097) | 0.131  (0.089) | -0.184^**^  (0.085) | -0.028  (0.086) | -0.178  (0.091) | 0.003  (0.094) |
| H_0_: No effect of university reform, Chow test p-value | 0.214 | | 0.001 | | 0.276 | | 0.050 | |
| Number of individuals | 308 | 402 | 308 | 402 | 448 | 334 | 448 | 334 |

*Note*: Age and location dummies included in all models. Standard errors in parentheses. *** significant at 1% level, ** significant at 5% level, * significant at 10% level
