# Supplementary material for: Are only-children different? Evidence from a lab-in-the-field experiment of the Chinese one-child policy
Source: PLoS One. 2022 Nov 8;17(11):e0277210. doi: 10.1371/journal.pone.0277210 (PMC9642884; doi:10.1371/journal.pone.0277210)
Supplement: S11 Table — (DOCX) [file pone.0277210.s011.docx]

**S11 Table. Regression model of time preferences to test effects of university reform**

|  | **Using observed status of university education** | | **Using predicted status of university education** | |
| --- | --- | --- | --- | --- |
|  | No univ. | Univ. | No univ. | Univ. |
| $\log\left( \beta\right)$ | -0.003  (0.003) | -0.002  (0.004) | -0.003  (0.003) | -0.002  (0.004) |
| $\log\left( \delta\right)$ | -0.010^***^  (0.000) | -0.010^***^  (0.000) | -0.010^***^  (0.000) | -0.010^***^  (0.000) |
| $\log\left( \beta\right)$ $\times$First stage OCP | -0.009^*^  (0.005) | -0.002  (0.005) | -0.005  (0.005) | -0.005  (0.006) |
| $\log\left( \delta\right)$ $\times$First stage OCP | -0.000  (0.001) | -0.000  (0.001) | -0.000  (0.001) | -0.001  (0.001) |
| $\log\left( \beta\right)$ $\times$Second stage OCP | -0.004  (0.004) | -0.004  (0.004) | -0.002  (0.004) | -0.006  (0.005) |
| $\log\left( \delta\right)$ $\times$Second stage OCP | -0.000  (0.001) | -0.001  (0.001) | -0.001^*^  (0.000) | -0.000  (0.001) |
| Number of observations | 1520 | 1608 | 1792 | 1336 |
| Number of individuals | 380 | 402 | 448 | 334 |
| H_0_: No effect of university reform Chow-test p-value | 0.002 |  | 0.616 |  |

*Note*: Age and location dummies included in all models. Clustered at individual level. Standard errors in parentheses. *** significant at 1% level, ** significant at 5% level, * significant at 10% level.
