# Supplementary material for: Are only-children different? Evidence from a lab-in-the-field experiment of the Chinese one-child policy
Source: PLoS One. 2022 Nov 8;17(11):e0277210. doi: 10.1371/journal.pone.0277210 (PMC9642884; doi:10.1371/journal.pone.0277210)
Supplement: S12 Table — (DOCX) [file pone.0277210.s012.docx]

**S12 Table. Regression models of behavioral experiments to test effects of university reform**

|  | **Using observed status of university education** | | | | | | | | | | | |
| --- | --- | --- | --- | --- | --- | --- | --- | --- | --- | --- | --- | --- |
|  | Public Good | | | | Competition | | | | Ultimatum | | | |
|  | Contribution | | Performance increase | | | Choose tournament | | Offer | | | Min. acceptable offer | |
|  | No univ. | Univ. | No univ. | Univ. | | No univ. | Univ. | No univ. | | Univ. | No univ. | Univ. |
| First stage OCP | -1.317  (0.995) | 0.480  (1.000) | -0.590  (0.471) | 0.769  (0.490) | | -0.174  (0.291) | 0.396  (0.273) | 0.446  (0.369) | | 0.215  (0.516) | -0.812  (1.192) | 0.613  (1.223) |
| Second stage OCP | -0.190  (1.652) | -2.025  (1.550) | -0.133  (0.841) | 1.152  (0.760) | | -0.649  (0.493) | 0.336  (0.427) | 0.818  (0.637) | | 0.770  (0.799) | -2.227  (2.060) | 1.287  (1.896) |
| University reform | 0.448  (1.243) | 0.484  (1.176) | 0.069  (0.613) | -0.728  (0.576) | | 0.483  (0.373) | -0.140  (0.320) | -0.500  (0.480) | | -0.743  (0.607) | 1.867  (1.550) | 1.530  (1.439) |
| H_0_: No effect of university reform, Chow test p-value | 0.064 | | 0.346 | | | 0.410 | | 0.643 | | | 0.207 | |
| Number of individuals | 380 | 402 | 380 | 402 | | 380 | 402 | 380 | | 402 | 380 | 402 |
|  | **Using predicted status of university education** | | | | | | | | | | | |
|  | No univ. | Univ. | No univ. | Univ. | | No univ. | Univ. | No univ. | | Univ. | No univ. | Univ. |
| First stage OCP | -1.274  (0.947) | 0.190  (1.017) | -0.494  (0.473) | 0.772  (0.483) | | -0.164  (0.287) | 0.381  (0.278) | 0.430  (0.396) | | 0.257  (0.514) | -0.425  (1.119) | 0.297  (1.219) |
| Second stage OCP | -0.751  (1.528) | -2.639  (1.690) | -0.096  (0.763) | 1.272  (0.803) | | -0.503  (0.459) | 0.142  (0.461) | 1.598  (0.639) | | 0.000  (0.855) | -0.200  (1.923) | -0.400  (2.027) |
| University reform | 0.746  (1.150) | 0.538  (1.272) | 0.321  (0.574) | -1.075  (0.604) | | 0.263  (0.348) | 0.149  (0.344) | -1.337  (0.481) | | 0.100  (0.643) | 1.123  (1.449) | 1.923  (1.523) |
| H_0_: No effect of university reform, Chow test p-value | 0.160 | | 0.054 | | | 0.029 | | 0.298 | | | 0.302 | |
|  | 448 | 334 | 448 | 334 | | 448 | 334 | 448 | | 334 | 448 | 334 |

*Note*: Age and location dummies included in all models Standard errors in parentheses. *** significant at 1% level, ** significant at 5% level, * significant at 10% level.
