# Supplementary material for: Are only-children different? Evidence from a lab-in-the-field experiment of the Chinese one-child policy
Source: PLoS One. 2022 Nov 8;17(11):e0277210. doi: 10.1371/journal.pone.0277210 (PMC9642884; doi:10.1371/journal.pone.0277210)
Supplement: S1 Text — (DOCX) [file pone.0277210.s013.docx]

**S1 Text. Description of experiments**

***Risk preferences***

In the uncertainty experiments, subjects made repeated choices between a fixed amount of money and a lottery, building on the design of Sutter et al. (2013). The uncertainty experiments included a risk and an uncertainty experiment, both of which had three tasks, and each task included 80 choices. Only one task, and only one choice of that specific task, was randomly selected to be played to determine how much money a subject would earn. We used a choice list where the subject chose repeatedly between a safe amount and a lottery with a certain probability of winning a fixed amount (e.g., Sutter et al., 2013).

In the risk experiment, subjects chose between a lottery with a chance to win 80 yuan and a safe amount of money increasing from 1 yuan to 80 yuan. The probability of winning was 50% in Task 1, 10% in Task 2, and 90% in Task 3. For each probability, subjects made repeated decisions between the lottery and the safe amount, with the safe amount increased by 1 yuan in each step. To make it a bit easier, subjects were instructed to draw a line between the rows where they started to prefer the safe amount.

The payout from the risk experiment was determined as follows (if the risk experiment had been randomly chosen to be payout relevant): If the subject chose the safe amount of money, the corresponding amount was paid. If the subject chose the lottery, the experimenter filled a red bag with 10 balls numbered from 1 to 10. Before drawing a ball, the subject told the experimenter five numbers between 1 and 10 in the case of a 50% probability of winning (one number if 10% and nine numbers if 90%), and then the subject drew a ball from the red bag. If the subject drew a ball with any of the chosen numbers, she received 80 yuan; otherwise, she did not receive anything.

The uncertainty experiment was similar to the risk experiment in that it also included three tasks with 80 choices in each task, but there was one important difference: The composition of 10 balls in a black bag was unknown. Subjects knew that a number between 1 and 10 was written on each ball, but not how many of each number were in the bag. The subjects needed to decide whether to draw a ball from the black bag with the chance of winning 80 yuan or to receive a safe amount of money increasing from 1 yuan to 80 yuan. Again, to make it easier, subjects were instructed to draw a line between the rows where they started to prefer the safe amount. As in the risk experiment, the three tasks had different winning probabilities of 10%, 50%, and 90%.

***Time preferences***

In the time preference experiments, Subjects were asked to make repeated choices between a sooner payment that was fixed and a later payment that would increase. The experiment included four tasks, and each task had 20 choices. Only one task, and only one choice of that specific task, was randomly selected to be played to determine how much money subjects would earn. The four tasks meant making decisions between (i) today and one week, (ii) one week and two weeks, (iii) today and two weeks, and (iv) two weeks and four weeks. In all the tasks, the sooner payment was 40 yuan, and the later payment increased from 41 yuan to 60 yuan. As in the risk and uncertainty experiments, subjects were instructed to draw a line between the rows where they started to prefer a later payment. The payment date differed depending on the decisive choice: If the subjects chose payment today, they were paid via bank transfer within two hours after the experiment. If they chose a later payment, subjects were also paid via bank transfer, but on the specific date they had chosen (one week, two weeks, or four weeks). Whether they had chosen a sooner or later payment, the subjects each received a slip of paper with the amount earned and the payment date, which was signed by the project leader.

***Competition experiment***

The competition experiment followed the design of Niederle and Vesterlund (2007), with three tasks, only one of which would be randomly selected as payoff relevant. Each subject was randomly matched with three other participants to form a group, but they did not know who the other members were. The group composition was the same during the whole competition experiment, and in each group, two were men and two were women. Subjects faced the task of calculating the sum of five randomly chosen two-digit numbers. Subjects were not allowed to use a calculator, but they could write the numbers down and make use of pens and scratch paper that we provided. So that subjects could familiarize themselves with the task, they first had a one-minute trial round. After that, the formal tasks started.

For Task 1, which had a piece rate payment, subjects were asked to calculate the sum of five randomly chosen two-digit numbers and write their answers on an answer sheet. The time for this task was three minutes. The payment was 3 yuan per problem solved if the task was randomly selected for payment. Task 2 was a tournament in which subjects had three minutes to solve the same type of math problems. Now the payment depended on the subject’s performance relative to that of other group members. The group member who solved the largest number of problems correctly received 12 yuan per problem solved, while the other participants received no payment. In case of a tie, the ranking between the members with equal performance was determined randomly. For Task 3, subjects first had to choose the payment schedule, piece rate or tournament, and then again solve the same type of math problems. If a subject chose the piece rate, payment was again 3 yuan per problem solved. If a subject chose tournament, her performance was evaluated relative to the performance of the other three group members in Task 2. If the subject solved more problems correctly than the other three group members, payment was 12 yuan per problem solved. Finally, subjects guessed their rank relative to other group members in Tasks 1 and 2. For each correct guess, they earned 3 yuan.

We needed to ensure that there were two men and two women in each group. Therefore, male and female subjects each drew a decision sheet from two separate boxes where each sheet had a letter from A to Z.^[[1]](#footnote-1)^ Two men and two women who had the same letter were placed in the same group. At the end of the experiment, each subject drew from a lottery with balls numbered 1 to 3 to decide which task would be used as the decisive task for payment.

***Public good experiment***

In the public good experiment, we use a design similar to that of Fischbacher et al. (2001), in which subjects made two contribution tasks. Each subject was endowed with 20 tokens, each token equivalent to 2 yuan. Subjects were in groups consisting of four members. They were asked to allocate the 20 tokens between a private account and a public account. The money in the private account was the subject’s own money. The money in the public account would be shared by all the group members. For each group member, the income from the public account was equal to the total amount of money put into the public account by all group members multiplied by 0.4. By choosing a marginal per capita return from the public good below one, we created the incentive to free ride, but since the return from the public good would exceed one if all four group members contributed, it was socially optimal for all subjects to contribute. Each subject’s total income was equal to the income from his private account plus his share of income from the public account. Before the decisions were made, we included three control questions to ensure that subjects understood how to calculate the total income. Only when subjects had answered all the control questions correctly were they allowed to continue with the formal decisions.

In Task 1, subjects decided how many of their tokens they wanted to put into the public account and into the private account. In Task 2, subjects decided how much to contribute to the public account conditional on a specific average contribution of the other group members. There were 21 possible average contributions that ranged from 0 to 20 tokens. Since subjects did not know beforehand the average contribution of the three other group members, they had to state their contribution for each of these potential average contributions by the other three group members. To make all choice incentives compatible, three group members were randomly selected for whom Task 1 was payout relevant. For the fourth subject, the average contributions of the other three members were used in the contribution table in Task 2 to determine the allocation to public good. Subjects did not know with whom they were matched or which of the tasks would be payout relevant at the time of making their allocation decisions. In practice, we determined the payoff from the game by having each subject draw a ticket from a box. Each ticket had a letter from A to Z,^[[2]](#footnote-2)^along with the number 1 or 2. The letter determined which group the subject would belong to, and the number indicated which decision would be used for payment. Then groups were formed as described above and payment was calculated accordingly.

***Ultimatum bargaining experiment***

Subjects were randomly matched in pairs for the ultimatum bargaining experiment, and again they did not have any information about each other. This experiment included two roles, player 1 and player 2. Subjects did not know their role beforehand, so they had to make decisions as both player 1 and player 2. After the experiment, the role of each subject was determined by rolling a two-sided die. The experiment worked as follows: Player 1 decided how to allocate an endowment of 40 yuan between the two subjects, and player 2 decided whether to accept or refuse the allocation. If player 2 accepted player 1’s allocation, then player 1 and player 2 split the money according to player 1’s allocation. If player 2 refused player 1’s allocation, then neither player received anything. The experiment consisted of four tasks: (i) In the role of player 1, the subject decided how to allocate the 40 yuan. (ii) In the role of player 2, the subject decided what would be the minimum amount she would accept. (iii) The subject guessed the average amount that all the other subjects allocated to player 2. (iv) The subject guessed the average minimum amount of all other subjects when responding as player 2. The payoff from the experiment was determined in a stepwise manner. First, each subject drew a ticket, which was labeled either 1 or 2, to determine whether the subject would be paid as player 1 or player 2. Then the subjects were matched in pairs. For each pair, the experimenter compared the decisions of player 1 and player 2. If player 1’s allocation was accepted by player 2, then both of them got the money according to player 1’s decision. Otherwise, both of them did not get anything.

1. After the 26 letters, we combined two different letters (AA, AB, AC, etc.). [↑](#footnote-ref-1)
2. Again, after the 26 letters, we combined two different letters (AA, AB, AC, etc.). [↑](#footnote-ref-2)
